# Estimating Clothing Thermal Insulation Using an Infrared Camera

**DOI:** 10.3390/s16030341

**Published:** 2016-03-09

**Authors:** Jeong-Hoon Lee, Young-Keun Kim, Kyung-Soo Kim, Soohyun Kim

**Affiliations:** 1 Department of Mechanical Engineering, KAIST, Daejeon 305-701, Korea; jhlee6@kaist.ac.kr (J.-H.L.); soohyun@kaist.ac.kr (S.K.); 2 Department of Mechanical and Control Engineering, Handong Global University, Pohang 791-708, Korea; ykkim@handong.edu

**Keywords:** clothing insulation, thermal comfort, PMV, infrared camera

## Abstract

In this paper, a novel algorithm for estimating clothing insulation is proposed to assess thermal comfort, based on the non-contact and real-time measurements of the face and clothing temperatures by an infrared camera. The proposed method can accurately measure the clothing insulation of various garments under different clothing fit and sitting postures. The proposed estimation method is investigated to be effective to measure its clothing insulation significantly in different seasonal clothing conditions using a paired *t*-test in 99% confidence interval. Temperatures simulated with the proposed estimated insulation value show closer to the values of actual temperature than those with individual clothing insulation values. Upper clothing’s temperature is more accurate within 3% error and lower clothing’s temperature is more accurate by 3.7%~6.2% error in indoor working scenarios. The proposed algorithm can reflect the effect of air layer which makes insulation different in the calculation to estimate clothing insulation using the temperature of the face and clothing. In future, the proposed method is expected to be applied to evaluate the customized passenger comfort effectively.

## 1. Introduction

Demands for smart and energy-efficient thermal systems that can control the air conditions according to the thermal comfort state of their users are increasing for home applications and vehicle Heating Ventilation and Air Conditioning (HVAC) systems. In electric vehicles, electric power to heat and blow warm air into the cabin in winter reduces mileage by nearly half. Therefore, it is important to satisfy thermal comfort as marginally as possible and control thermal comfort independently to save power according to the passenger number and position. In the same environment, different users (passengers) may experience different thermal comfort depending on their body temperature, clothing insulation, relative humidity, and other factors. Thus, a smart thermal comfort system is required to control climatic parameters operations such as air temperature, air flow direction, and air speed by sensing the complex thermal state of each passenger and the environment.

The standard measure of thermal comfort is the predicted mean vote (PMV) that is defined in ISO 7730 [1]. The PMV is calculated from a complex relationship of six input variables: air temperature, mean radiant temperature, air velocity, air humidity, clothing insulation, and metabolic rate of the passenger. If all six variables of the PMV could be measured in real time, then control of PMV for individual passengers could be realized. However, the variables of metabolic rate and clothing insulation cannot be detected by simple sensors: they are too complex and related to physical processes. Also, the compact space in a vehicle cabin and cost-effectiveness are major obstacles to realizing such a smart vehicular thermal system.

Some researchers have proposed various numerical models of clothing thermal insulation (hereafter referred to as clothing insulation). The commonly applied clothing thermal models are the simplified one-parameter model, two-parameter models, and three-parameter models [2]. The one-parameter model simply estimates the thermal behavior of clothing based on its dry thermal insulation [2]. Two-parameter models consider the additional factor of vapor permeability, and three-parameter model includes the air layer between the skin and clothing [2]. Other researchers have analyzed human temperature distribution, thermal sensation, comfort, and clothing insulation using more complex numerical methods than the above-mentioned simple models, that solve thermal interaction between the environment and a virtual thermal mannequin based on a human thermal physiological model [3]. However, although these methods offer accuracy, they require high computational power in a costly and time-consuming process.

The standard methods to measure clothing insulation use thermal mannequins, but a study by Konarsk *et al.* [4] reported that analyzing clothing insulation measured on human subjects yielded different results. Their paper analyzed the results of comparative clothing thermal insulation measurements determined in tests on volunteers and on a thermal mannequin under specified conditions, *i.e.*, a standing man and a standing mannequin in a climate chamber under wind-free conditions with three ensembles of disposable medical clothing. The clothing insulation on human subjects was found to be up to 13% higher than that measured on the thermal mannequin. Also, the measurement error on the mannequin was about 2%, whereas the measurement error on human subjects was as large as 18%. It can be inferred that characterizing actual clothing insulation on humans requires a more complex model, in which additional factors such as clothing fit and posture should be considered.

The effects of clothing fit and posture on human thermal condition were studied by Kakitsuba [5]. The study showed that the clothing fit (tight or loose fitting) and the posture of the human subject (standing, sitting on a floor, or sitting on a chair) are significant factors in the clothing area factor, that is the ratio of the clothing surface area to the body surface area. The clothing fit and the posture change the volume of the clothing micro-environment, which affects the clothing insulation.

Because the conventional measurement method with human subjects or with a thermal mannequin requires the same laboratory conditions, including various types of apparatus and specific facilities, which are cost-intensive, other researchers proposed simpler estimating clothing insulation based on a regression analysis that relates some variables to the clothing insulation obtained from experimental data. A study by McCullough *et al.* proposed a regression analysis to predict the clothing insulation in terms of the body surface area covered by a single clothing layer, the body surface area not covered by clothing and the total clothing weight [6]. A study by Kwon *et al.* proposed that the clothing insulation can be predicted based on a multivariate linear regression model that incorporates the factors of clothing microclimate temperature, total weight of clothing, and upper clothing layers from a test [7]. However, clothing fit and posture effects were not considered and some parameters, that is, the weight of clothing and the number of clothing layers were determined manually prior to conducting the experiments. Despite the proposed methods of predicting clothing insulation, some parameters of them are still based on variables that are not directly measurable by a sensor in a real-time application and as long as models are applied to predict clothing insulation (including physical and virtual models), there won’t be a perfect prediction of the individual clothing insulation.

On the other hand, infrared thermography has shown great applications in clothing comfort research due to its direct and non-invasive measurement advantages [8]. Gasi and Bittencourt used infrared thermography to evaluate textile materials and it was found that if the temperature variation from thermal image is less, it gives higher thermal efficiency [9]. Matusiak mentioned in his article that irrespective of the thermal resistance of clothing material, a key role in heat exchange is played by the size of air layers closed between the human body and clothing surface, as well as between the particular layers of clothing, which shows a thermogram from an infrared camera [10].

Thus, this paper proposes a novel algorithm for estimating clothing insulation with parameters that can be directly and in real time measured by an IR camera. First, this paper derives the correlation formula between the clothing insulation and the skin and clothing temperatures. It also investigates the effects of variables such as clothing type and fit, that is, the air layer volume underneath the clothing. Then, the proposed algorithm is validated by conducting experiments on human subjects.

## 2. Algorithm of Estimating Clothing Insulation

The our idea is to automatically estimate the manually defined parameter up to now, clothing insulation, using sensor technology (infrared camera) to assess thermal comfort. In this section, a novel algorithm for estimating clothing insulation will be based on the non-contact and real-time measurements of the face and clothing temperatures by an infrared camera to measure accurately the clothing insulation of various garments under different clothing fit and sitting postures. The reason that we can do that is that the face and clothing temperatures will be a powerful footprint which is thermally interactive between environment and human body like as window, its interactive level means clothing insulation. We will derive from static definition to dynamic field with correction factor reflecting air velocity effects.

### 2.1. Under Static Thermal State

It is assumed that the clothing on the human body is at a steady thermal state and dry heat transfer condition. Total static insulation is the thermal insulation from the body surface to the environment including all clothing, enclosed air layers, and the boundary air layer. Static clothing insulation is the thermal insulation from the skin surface to the outer clothing surface including enclosed air layers under static conditions, that can be expressed as Equation (1) [11]:

(1) $$I_{tot\_st}=I_{cl\_st}+\frac{I_{a\_st}}{f_{cl}}$$

where, *I_cl_st_* is the static clothing insulation, *I_a_st_* is the static air thermal resistance and *f_cl_* is the clothing area factor, that is the ratio of the clothed and naked-skin surface areas. It can be calculated in terms of the static clothing insulation (*I_cl_st_*) [11], as:

(2) $$f_{cl}=1+1.97I_{cl\_st}$$

Unit of thermal insulation value of clothing leads to Equation (3):

(3) $$I_{cl\_st}[m^{2}K/W]=0.155\cdot I_{cl}[\text{clo}]$$

Then, the total static insulation of Equation (1) can be re-expressed in terms of *I_cl_* from Equation (3):

(4) $$I_{tot\_st}=0.155I_{cl}+\frac{I_{a\_st}}{1+0.305I_{cl}}$$

### 2.2. Under Dynamic Thermal State

In actual applications, such as inside a vehicle cabin with air ventilation, the thermal state is dynamic. When there is movement of the air and the human subject, the total thermal insulation may be decreased. Thus, in the dynamic thermal state, the dynamic air insulation (*I_a_dyn_*) and the dynamic total clothing insulation (*I_tot_dyn_*) must be calculated, that can be obtained by applying correction factors of *C_orr,ia_* and *C_orr,tot_*, respectively, to the static conditions [11,12], as expressed by:

(5) $$\begin{array}{l} I_{a\_dyn}=\;C_{orr,ia}I_{a\_st}\\ I_{tot\_dyn}=C_{orr,tot}I_{tot\_st} \end{array}$$

(6) $$C_{orr,tot}=\{\begin{array}{cc} C_{orr,cl} & \text{for}\;I_{cl}\geq 0.6\\ \frac{(0.6-I_{cl})C_{orr,ia}+I_{cl}C_{orr,cl}}{0.6} & \text{for}\;I_{cl}<0.6 \end{array}$$

(7) $$\begin{array}{l} C_{orr,ia}=e^{\left\{-0.559(v_{ar}-0.15)+0.057{\left(v_{ar}-0.15\right)}^{2}+0.271v_{w}-0.027{v_{w}}^{2}\right\}}\leq 1.0\\ C_{orr,cl}=e^{\left\{-0.263(v_{ar}-0.15)+0..0272{\left(v_{ar}-0.15\right)}^{2}-0.193v_{w}+0.101{v_{w}}^{2}\right\}}\leq 1.0 \end{array}$$

The dynamic clothing insulation are considered as Equation (6) according to clothing insulation range using the definition in [11,12] and recent empirical correction factors from 486 thermal mannequin tests in totally nine different testing conditions regarding air velocity (*v_ar_*) and human walking velocity (*v_w_*) as Equation (7) [13]:

(8) $$I_{cl\_dyn}=I_{tot\_dyn}-\frac{I_{a\_dyn}}{f_{cl}}=\frac{t_{sk}-t_{cl}}{C+R}$$

The convection (*C*) and radiation (*R*) heat exchange between the skin and the clothing for a seated subject are calculated from:

(9) $$C+R=f_{cl}\left\{h_{c}(t_{cl}\;-t_{a})+3.85\times 10^{-8}\;\left({t_{cl}}^{4}\;-\;{{\bar{t}}_{r}}^{4}\right)\right\}$$

where the 3.85 value comes from the assumption that the emissivity value of a seated passenger is 0.97 and the fraction of skin surface involved in heat exchange by radiation is equal to 0.70 for a seated subject.

Combining Equations (8) and (9), the dynamic clothing insulation (*I_cl_dyn_*) can be expressed as Equation (10) using α, that is defined as the ratio of the temperature difference between the skin and the clothing to convection (*C*) and radiation (*R*) heat exchange between the skin and the environment:

(10) $$\begin{array}{l} I_{cl\_dyn}=\frac{t_{sk}-t_{cl}}{C+R}=\frac{1}{f_{cl}}\left\{\frac{t_{sk}-t_{cl}}{h_{c}(t_{cl}\;-t_{a})+3.85\times 10^{-8}\left(t_{cl}{\;}^{4}\;-\;{{\bar{t}}_{r}}^{4}\right)}\right\}=\frac{\alpha }{f_{cl}}\\ where,\;\alpha =\frac{t_{sk}-t_{cl}}{h_{c}(t_{cl}\;-t_{a})+3.85\times 10^{-8}\left(t_{cl}{\;}^{4}\;-\;{{\bar{t}}_{r}}^{4}\right)} \end{array}$$

### 2.3. Generalized Clothing Insulation Formula under Static And Dynamic States

This paper proposes a method to derive *I_cl_* to enter into the thermal comfort index (PMV) formula using measurable or calculated parameters at dynamic conditions.

Equation (1) can be rearranged in terms of *I_cl_st_* by substituting Equation (5):

(11) $$I_{cl\_st}=I_{tot\_st}-\frac{I_{a\_st}}{f_{cl}}=\frac{I_{tot\_dyn}}{C_{orr,cl}}-\frac{I_{a\_st}}{f_{cl}}$$

Also, Equation (7) can be rearranged in terms of the total dynamic heat resistance (*I_tot_dyn_*):

(12) $$I_{tot\_dyn}=\frac{t_{sk}-t_{cl}}{C+R}+\frac{I_{a\_dyn}}{f_{cl}}$$

Substituting Equation (12) into Equation (11), *I_cl_st_* becomes:

(13) $$I_{cl\_st}=\frac{1}{C_{orr,tot}}\left(\frac{t_{sk}-t_{cl}}{C+R}+\frac{I_{a\_dyn}}{f_{cl}}\right)-\frac{I_{a\_st}}{f_{cl}}$$

Using the variable α from Equation (10), *I_cl_st_* can be expressed as:

(14) $$I_{cl\_st}=\frac{1}{C_{orr,tot}}\left(\frac{\alpha }{f_{cl}}+\frac{I_{a\_dyn}}{f_{cl}}-\frac{C_{orr,tot}I_{a\_st}}{f_{cl}}\right)=\frac{1}{C_{orr,tot}f_{cl}}\left(\alpha +I_{a\_dyn}-C_{orr,tot}I_{a\_st}\right)$$

Next, the dynamic air insulation is substituted by the simpler static air insulation *i.e*., $I_{a\_dyn}=\;C_{orr,ia}I_{a\_st}$ to express *I_cl_st_* as:

(15) $$I_{cl\_st}=\frac{1}{C_{orr,tot}f_{cl}}\left\{\alpha +I_{a\_st}\left(C_{orr,ia}-C_{orr,tot}\right)\right\}$$

The final step is expressing Equation (15) in terms of the clothing insulation by using Equations (2) and (3) to obtain:

(16) $$I_{cl}(1+0.305I_{cl})=\frac{1}{0.155C_{orr,tot}}\left\{\alpha +I_{a\_st}\left(C_{orr,ia}-C_{orr,tot}\right)\right\}$$

**Figure 1:**
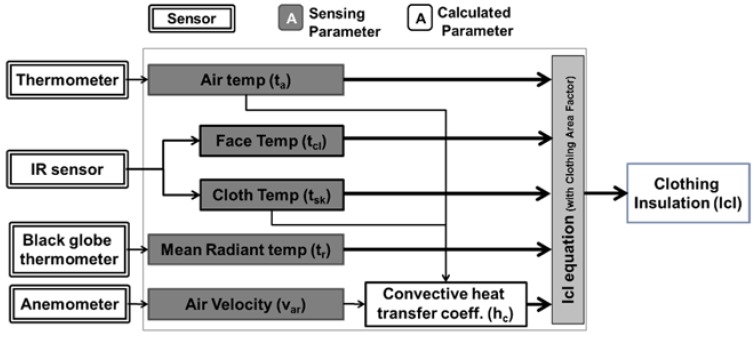
Proposed clothing insulation calculation block diagram.

Therefore, the newly proposed algorithm can estimate clothing insulation using a non-linear function of the exposed skin temperature (*t_sk_*), the clothing temperature (*t_cl_*) and the surrounding thermal condition, air temperature (*t_a_*), mean radiant temperature (*t_r_*), relative air velocity (*v_ar_*) with the form of $I_{cl}(t_{sk},t_{cl},t_{a},t_{r},v_{ar})=0$ expressed as

(17) $$0.305{I_{cl}}^{2}+I_{cl}-\frac{1}{0.155C_{orr,tot}}\left\{\alpha +I_{a\_st}\left(C_{orr,la}-C_{orr,tot}\right)\right\}=0\;\begin{array}{l} where,\;\alpha =\frac{t_{sk}-t_{cl}}{h_{c}(t_{cl}\;-t_{a})+3.85\times 10^{-8}\left(t_{cl}{\;}^{4}\;-\;{{\bar{t}}_{r}}^{4}\right)}\\ h_{c}=\{\begin{array}{c} 2.38{|t_{cl}-t_{a}|}^{0.25}\;for\;2.38{|t_{cl}-t_{a}|}^{0.25}\geq \;12.1\sqrt{v_{ar}}\\ 12.1\sqrt{v_{ar}}\;for\;2.38{|t_{cl}-t_{a}|}^{0.25}<\;12.1\sqrt{v_{ar}} \end{array}\; \end{array}\;\begin{array}{l} \;C_{orr,tot}=\{\begin{array}{cc} C_{orr,cl} & \text{for}\;I_{cl}\geq 0.6\\ \frac{I_{cl}(C_{orr,cl}-C_{orr,ia})}{0.6}+C_{orr,ia} & \text{for}\;I_{cl}<0.6 \end{array}\\ C_{orr,ia}=e^{\left\{-0.559(v_{ar}-0.15)+0.057{\left(v_{ar}-0.15\right)}^{2}+0.271v_{w}-0.027{v_{w}}^{2}\right\}}\leq \;1.0\\ C_{orr,cl}=e^{\left\{-0.263(v_{ar}-0.15)+0..0272{\left(v_{ar}-0.15\right)}^{2}-0.193v_{w}+0.101{v_{w}}^{2}\right\}}\;\leq \;1.0 \end{array}$$

where *C_orr,ia_* and *C_orr,cl_* are the correction factors for air velocity (*v_ar_*) and human walking velocity (*v_w_*) and α is the temperature gradient between the skin and the clothing surface divided by the dry heat loss (convection and radiation) per unit of nude body surface area. Figure 1 shows a novel input/output block diagram for estimating the clothing insulation with parameters that can be directly measured by an infrared (IR) camera. However, this proposed calculation equations have to be applied carefully considering some limitation of steady-state condition, not transient or dynamic changing condition because this proposed algorithm are derived from steady-state form of the energy conservation equation. If IR camera doesn’t have the function to assign each emissivity to the face and clothing region to get accurate temperature, the emissivity of the clothing has to be almost similar to that of face skin.

## 3. Experiments, Simulation and Analysis

### 3.1. Experiments: Comparison of the Clothing Insulation between Estimated on the Proposed Algorithm and Calculated According to ISO 9920 and Its Measurement

Three clothing ensembles were selected in order to verify the proposed clothing insulation algorithm. They represent typical seasonal working clothing at office, that is, summer, spring/fall and winter as follows and whose insulations are listed on Table 1.

**Figure 2:**
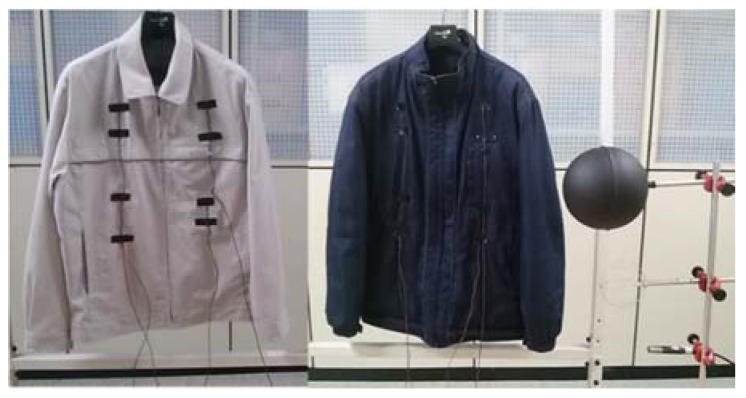
Spring/fall and winter working jacket uniform.

In particular experiments were conducted with working jacket uniforms which had same materials and design to improve accuracy as shown in Figure 2:

- Winter clothing ensembles: underwear pants briefs, trousers straight fitted, socks, shoes, underwear shirts sleeveless, shirts long sleeves shirt collar, winter working jacket (company’s uniform).
- Spring/fall clothing ensembles: underwear pants briefs, trousers straight fitted, socks, shoes, underwear shirts sleeveless, shirts long sleeves shirt collar, spring/fall working jacket (company’s uniform).
- Summer clothing ensembles: underwear pants briefs, trousers straight fitted, socks, shoes, underwear shirts sleeveless, shirts long sleeves shirt collar

Experiments were carried out on sitting posture at rest in the climatic room during each 30 min exposure under physical characteristics of subjects and environmental conditions measured by SWEMA, ISO 7726, 7730 measurement equipment as shown in Table 2, and thermal images were recorded by a ThermaCAM S45 thermographic camera (FLIR, Wilsonville, OR, USA) with an infrared resolution of 320 × 240 pixels, spatial resolution 1.3 mrad, thermal sensitivity below 0.05 °C, and accuracy of ±2 °C.

Table 3, Table 4 and Table 5 show the results of thermal images, the measured temperature, and estimated upper, lower and ensemble clothing insulation in each ensemble.

But for the same lower clothing ensembles, winter lower clothing insulation measured equally to spring/fall lower clothing insulation as *t*-value = 2.32, *p*-value = 0.059, as well as, spring/fall lower clothing insulation is measured equally to summer lower clothing insulation as *t*-value = 1.68, *p*-value = 0.154.

**Figure 3:**
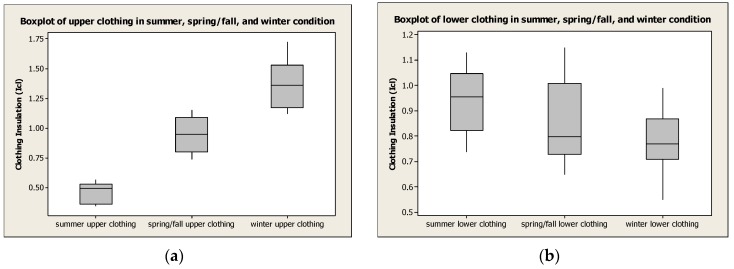
Boxplot for the estimated clothing insulation in the case of summer, spring/fall, and winter clothing ensembles using the proposed estimation method. (**a**) Boxplot for the estimated upper clothing insulation; (**b**) Boxplot for the estimated lower clothing insulation.

In conclusion, the proposed estimation method is effective to measure its clothing insulation significantly in these different clothing ensembles as shown in Figure 3. Next investigation on accuracies of the proposed method will be analyzed using simulation stated in following paragraph with the comparison between resultant temperatures applied estimated insulation values and those of reference insulation values.

The purpose of these experiments was to investigate whether the proposed estimation method is effective for measuring clothing insulation under different clothing conditions and to show how accurate the proposed method is compared with previous reference values. Therefore reference values are necessary and the reference insulation for an ensemble was obtained that based on a summation of the insulation of individual garments using the empirical equation, Equation (18) [14]

(18) $$I_{cl}=0.161+0.835\sum I_{clu}$$

Table 6 shows the summarized results of the estimated clothing insulation, reference clothing insulation, and errors for ensembles and subjects. In order to analyze whether the proposed estimation method can measure different clothing insulation on the same subject or not, experiments were carried out between paired subjects, that is, three clothing ensembles tested on one subject independently. Therefore, paired t-test was applied in statistics using Minitab Release 14. After checking the normality of all samples (upper and lower clothing insulation in three ensembles, six samples in total) with an Anderson-Darling test (*p* > 0.05), a paired t-test was applied to analyze the measurability of different insulation despite their variances. A coefficient of significance α = 0.01 (99% confidence interval) was adopted. Paired t for summer upper clothing—spring/fall upper clothing with null hypothesis t-test of mean difference = 0 (*vs.* < 0) shows *t*-value = −6.77, *p*-value = 0.001. This analysis reveals that it is not plausible that the mean difference = 0, and so we can conclude that spring/fall upper clothing insulation can be measured better than summer upper clothing insulation using the proposed estimation method. Also, we can conclude that winter upper clothing insulation can be measured better than spring/fall upper clothing insulation using the proposed estimation method as *t*-value = −4.63, *p*-value = 0.002.

### 3.2. Simulation: Comparison of Regional Temperature between Actual and Simulated with the Estimated Clothing Insulation Value and the Reference Clothing Insulation Value

Although standard values of clothing insulation are described in ISO 9920 [15], they may be not a suitable reference for our experiments with sitting human subjects. The clothing insulation values in ISO 9920 are only for the standing posture and did not considered the fit of the clothing, especially as it pertains to upper clothing and the air layer between clothing that are crucial parameters for clothing insulation [8]. In addition, although the insulation of an ensemble calculated from the individual garment (Equation (18)) gives acceptable accuracy for typical indoor clothing, overall accuracies are on the order of ± 25% [16].

**Figure 4:**
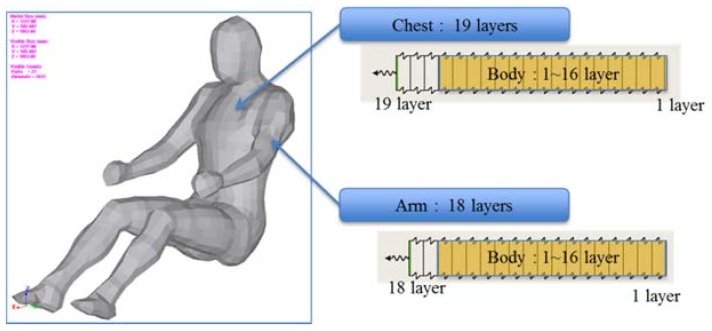
A simulation model with various layers of clothing and body segments in the case of winter ensemble. Tested insulation values are obtained from ISO 9920 and measurements.

Thus, a simulation was conducted to analyze the accuracy of the proposed algorithm for estimating clothing insulation applied to a sitting person by comparing simulated temperature distribution with actual one. The thermal analysis software is PowerTherm that is based on the UC Berkeley Thermo-physiological Comfort Model (BTCM) which developed by Huizenga *et al.* and has been validated against empirical physiological responses in transient, non-uniform thermal environments [3,17,18]. The human model for the simulation was created by applying a standard height (172 cm) and weight (72.4 kg), male gender, body fat of 14.95 kg, skin emissivity of 0.95, absorptivity of 0.72 (brown skin), and a basal metabolic rate of 0.8 met, to be closely related to the test human subject as shown in Table 2. The winter ensemble simulation model is designed to be wearing three layers of clothing with a winter working jacket as the outermost layer, similar to the test subject’s clothing as shown in Figure 4.

The simulation program can estimate the expected temperature of the skin and clothing of the model when the test conditions and clothing insulation values are used as input parameters. Thus, the simulation model can be evaluated what insulation value is more accurate by comparing the estimated temperature of the skin and clothing with temperature measurements by an IR camera. For the purpose, a test setup was designed with the parameter values shown in Table 7, Table 8 and Table 9 on a test subject. The temperatures of the face and clothing were measured using an IR camera. The clothing insulation *I_cl_* is calculated using the proposed algorithm.

The same test conditions were used as the input parameters in the simulation program to estimate the resulting clothing temperature (chest, legs) and face temperature_._ We analyzed two simulation models with different clothing insulation conditions:

Case 1: Individual clothing insulation values without air layers between clothing.

Thermal resistance values for each clothing layer are from ISO 9920 and measured data. The air insulation layer between clothing is not considered.

Case 2: Ensemble thermal insulation values from the proposed clothing insulation Equation.

The estimated clothing insulation is used as the ensemble thermal insulation values based on the proposed clothing insulation estimation.

As shown in Table 10, Table 11 and Table 12, when the clothing insulation calculated by the proposed algorithm was used in the simulation mode, as in Case 2, temperatures simulated with the estimated insulation value were closer to the values of actual temperature than individual clothing insulation values without air layers between clothing, as in Case1. Compared with previous reference insulation values, the % face temperature error was almost same within 1% between cases, but the chest clothing temperature applying the proposed insulation value was more accurate with an 3% error reduction shown as Δ% error in Table 10, Table 11 and Table 12 and legs’ clothing temperature applying the proposed insulation value was more accurate with a 3.7%~6.2% error reduction. Therefore, it can be concluded that the proposed algorithm is more accurate than one using the previous measured individual garment insulation database for these clothing ensemble sets.

### 3.3. Experiment with the Effect Of Air Layer Thickness

We supposed that the clothing insulation differences between the estimated value and reference value are caused by the effect of air layer between clothing because the clothing fit (tight or loose fitting) and the posture of the human subject (standing or sitting on a chair) are such a significant factors which can change the volume of the clothing micro-environment (air insulation).

To verify whether the proposed algorithm can reflect the air layers effect or not, an experiment was conducted with several different thicknesses of air layers on the test subject. Depending on the fit of the clothing, different thicknesses of air layers can affect the clothing insulation. To simulate the thickness of air layers, air cap films were wrapped around the upper body as shown in Table 13. For each test, the seated human subject wore the same pants but with different air layer thickness under a tight-fitting long-sleeved shirt.

The experimental results show that the difference of face and clothing temperature (*t_sk_ − t_cl_*) increases as the thickness of air layer increases. This means that the air layer thickness is closely correlated with the temperature difference between the face and clothing. Thus, the estimation of the clothing insulation that includes the effect of air layer can be made using the temperature of the face and clothing as in the proposed algorithm. To show whether the proposed algorithm can reflect the effect of different air layer thicknesses, the estimated *I_cl_* values were inserted into the simulation model. The simulated temperature of the skin and clothing due to the estimated clothing insulation were compared with the actual temperature measured by the IR camera.

As Table 14 shows, the simulation output has less than 1.9% error relative to the actual temperature. Thus, it can be concluded that the proposed algorithm can reflect the effect of different air layer thickness in the calculation to estimate clothing insulation. The relationship between difference of temperature (*t_sk_ − t_cl_*) and clothing insulation according to air layer thickness is judged by the correlation factor R^2^, R^2^ is 0.89 in linear regression model. It means that the difference of temperature (*t_sk_ − t_cl_*) is related with clothing insulation. In the average skin (face) temperature, it was of little importance whether you wear glasses or not if the region of interest may be assigned as face’s area below glasses as in Table 13.

## 4. Conclusions

In this paper, a novel algorithm for estimating the clothing insulation using an IR camera is proposed. The proposed algorithm can estimate clothing insulation values in real time from a relationship of the exposed skin temperature, and the clothing temperature, that are all directly measurable by an IR camera. To investigate whether the proposed estimation method is effective to measure clothing insulation accurately for different clothing conditions, experiments using three clothing ensembles on one subject independently were performed. Paired t-test was applied with a coefficient of significance α = 0.01 (99% confidence interval). We can conclude that spring/fall upper clothing insulation can be measured greater than summer upper clothing insulation using the proposed estimation method. Also, we can conclude that winter upper clothing insulation can be measured greater than spring/fall upper clothing insulation using the proposed estimation method, but lower clothing insulation is measured equally for three different ensembles as same lower clothing ensembles.

Temperatures simulated with the estimated insulation value were closer to the actual temperature values than temperatures simulated with individual clothing insulation values without air layers between clothing. The upper clothing’s surface temperature was more accurate with a 3% error reduction and the lower clothing’s surface temperature was more accurate with a 3.7% ~ 6.2% error reduction. Therefore, it can be concluded that the proposed algorithm is more accurate than those using the previous measured individual garment insulation database.

The estimation of the clothing insulation that includes the effect of air layer can be made using the temperature of the face and clothing as in the proposed algorithm. The simulation output has less than 1.9% error relative to the actual temperature. Thus, it can be concluded that the proposed algorithm can reflect the effect of different air layer thickness in the calculation to estimate clothing insulation.

Thus, the proposed algorithm accurately reflects the effects of the different clothing to estimate the clothing insulation by real-time and non-contact measurements of face and clothing temperatures. Future work should focus on developing a specialized formula that reflects cabin air conditioning characteristics through a variety of vehicle tests. This could eventually lead to methods for assessing thermal comfort in vehicle cabins and applications in customized passenger comfort control.

## Figures and Tables

**Table 1:** Tested garments and their thermal insulation from ISO 9920 and measurement (* value measured by methods ASTM 1518 or equivalent).

Ensembles	Thermal Insulation	
	clo	m^2^·KW^−1^
Underwear pants briefs	0.04	0.006
Trousers straight fitted	0.22	0.034
Socks	0.02	0.003
Shoes	0.03	0.003
Underwear shirts sleeveless	0.06	0.009
Shirts long sleeves shirt collar	0.31	0.048
Spring/fall working jacket	0.29 *	0.045 *
Winter working jacket	0.47 *	0.073 *

**Table 2:** Mean values and standard deviations (SD) of physical characteristics of subjects and environmental conditions concerning clothing ensembles tested on sitting at rest.

Clothing Ensemble	Sex	Age ± SD (year)	Height ± SD (cm)	Mass ± SD (kg)	*t_r_* ± SD (Radiant temp) (°C)	*t_a_* ± SD (Ambient temp) (°C)	*v_ar_* ± SD (Air Velocity) (m/s)	*Humidity* ± SD (%RH)
Winter	male (n = 7)	34.3 ± 1.4	173.9 ± 6.1	73.1 ± 8.5	22.12 ± 0.28	21.76 ± 0.60	0.06 ± 0.06	20.04 ± 0.45
Spring/Fall	male (n = 7)	34.3 ± 1.4	173.9 ± 6.1	73.1 ± 8.5	22.85 ± 0.19	22.41 ± 0.42	0.06 ± 0.06	19.74 ± 0.38
Summer	male (n = 6)	34.7 ± 1.0	173.7 ± 6.7	71.5 ± 8.0	23.40 ± 0.15	22.79 ± 0.50	0.07 ± 0.05	18.94 ± 0.42

**Table 3:** Results of measured mean temperature and estimated clothing insulation concerning winter clothing ensembles after 30 minutes of exposure.

Thermal Images		Measured Mean Temperature			Estimated Clothing Insulation		
Upper clothing	Lower clothing	*t_cl_up_* ± SD (upper cloth) (°C)	*t_cl_lo_* ± SD (lower cloth) (°C)	*t_sk_* ± SD (face) (°C)	*Icl_up_esti* (upper clothing) (clo)	*Icl_lo_esti* (lower clothing) (clo)	*Icl_esti* (Ensemble clothing) (clo)
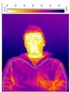	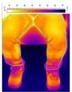	25.03 ± 0.81	27.83 ± 0.06	32.77 ± 0.4	1.36	0.55	1.76
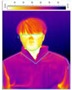	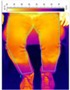	24.65 ± 0.08	26.91 ± 0.08	32.50 ± 0.08	1.53	0.71	2.03
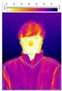	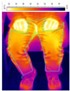	24.93 ± 0.05	26.96 ± 0.06	33.18 ± 0.05	1.46	0.77	2.02
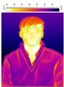	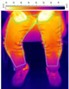	24.47 ± 0.05	26.09 ± 0.06	33.10 ± 0.05	1.72	0.99	2.43
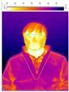	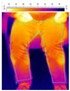	25.33 ± 0.06	26.33 ± 0.07	32.57 ± 0.06	1.20	0.87	1.89
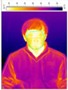	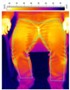	25.83 ± 0.06	27.03 ± 0.10	33.53 ± 0.12	1.12	0.78	1.75
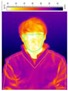	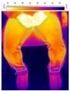	25.68 ± 0.10	27.10 ± 0.09	33.42 ± 0.04	1.17	0.76	1.77
Total (Mean ± SD)		25.13 ± 0.51	26.89 ± 0.56	33.01 ± 0.41	1.37 ± 0.22	0.78 ± 0.14	1.95 ± 0.24

**Table 4:** Results of measured mean temperature and estimated clothing insulation concerning spring/fall clothing ensembles after 30 min of exposure.

Thermal Images		Measured Mean Temperature			Estimated Clothing Insulation		
Upper clothing	Lower clothing	*t_cl_up_* ± SD (upper cloth) (°C)	*t_cl_lo_* ± SD (lower cloth) (°C)	*t_sk_* ± SD (face) (°C)	*Icl_up_esti* (upper clothing) (clo)	*Icl_lo_esti* (lower clothing) (clo)	*Icl_esti* (Ensemble clothing) (clo)
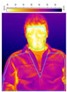	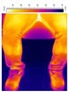	25.77 ± 0.35	27.83 ± 0.67	33.10 ± 0.26	1.15	0.65	1.66
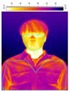	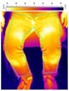	27.55 ± 0.07	27.59 ± 0.14	33.35 ± 0.07	0.74	0.73	1.38
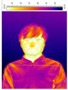	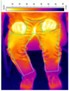	27.25 ± 0.07	27.88 ± 0.06	34.50 ± 0.00	0.93	0.77	1.63
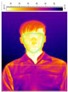	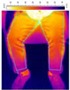	26.55 ± 0.13	26.80 ± 0.11	34.03 ± 0.06	1.09	1.01	1.68
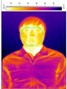	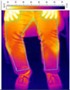	27.37 ± 0.06	26.23 ± 0.07	33.57 ± 0.15	0.80	1.15	1.81
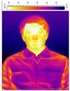	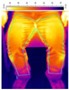	26.43 ± 0.25	26.71 ± 0.13	33.23 ± 0.12	0.96	0.95	1.76
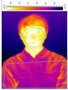	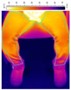	27.00 ± 0.14	27.58 ± 0.09	34.03 ± 0.06	0.95	0.80	1.61
Total (Mean ± SD)		26.93 ± 0.51	27.23 ± 0.64	33.69 ± 0.51	0.93 ± 0.15	0.87 ± 0.18	1.65 ± 0.14

**Table 5:** Results of measured mean temperature and estimated clothing insulation concerning summer clothing ensembles after 30 min of exposure.

Thermal Images		Measured Mean Temperature			Estimated Clothing Insulation		
Upper clothing	Lower clothing	*t_cl_up_* ± SD (upper cloth) (°C)	*t_cl_lo_* ± SD (lower cloth) (°C)	*t_sk_* ± SD (face) (°C)	*Icl_up_esti* (upper clothing) (clo)	*Icl_lo_esti* (lower clothing) (clo)	*Icl_esti* (Ensemble clothing) (clo)
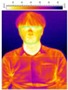	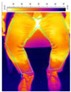	29.15 ± 0.13	27.91 ± 0.06	33.60 ± 0.27	0.49	0.74	1.18
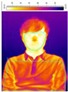	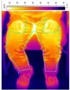	29.58 ± 0.22	27.80 ± 0.32	34.48 ± 0.21	0.50	0.85	1.29
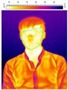	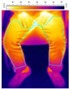	30.05 ± 0.07	27.02 ± 0.07	33.63 ± 0.06	0.35	0.99	1.28
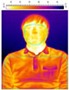	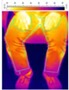	29.90 ± 0.10	26.59 ± 0.06	33.53 ± 0.21	0.37	1.13	1.41
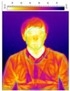	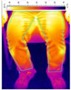	29.10 ± 0.22	27.00 ± 0.15	33.85 ± 0.24	0.52	1.02	1.44
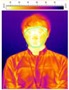	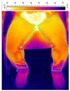	28.93 ± 0.12	27.38 ± 0.13	34.03 ± 0.21	0.57	0.92	1.40
Total (Mean ± SD)		29.45 ± 0.46	27.28 ± 0.51	33.85 ± 0.36	0.46 ± 0.09	0.94 ± 0.14	1.34 ± 0.10

**Table 6:** Comparison of clothing insulations between estimated values and reference values.

Clothing Ensemble	Estimated Clothing Insulation			*Icl_ref* (Reference Icl) (clo)	Error (*Icl_esti-Icl_ref*) (clo)
	*Icl_up_esti* ± SD (Upper Clothing) (clo)	*Icl_lo_esti* ± SD (Lower Clothing) (clo)	*Icl_esti* ± SD (Ensemble Clothing) (clo)		
Winter	1.37 ± 0.22	0.78 ± 0.14	1.95 ± 0.24	1.12	0.83
Spring/fall	0.93 ± 0.15	0.87 ± 0.18	1.65 ± 0.14	0.97	0.68
Summer	0.46 ± 0.09	0.94 ± 0.14	1.34 ± 0.10	0.73	0.61

**Table 7:** Simulation input parameters concerning each clothing ensembles (ensemble clothing insulation values estimated from mean values of environmental conditions).

Ensemble	*t_r_* (°C)	*t_a_* (°C)	*t_cl_up_* (°C)	*t_cl_lo_* (°C)	*t_sk_* (°C)	*v_ar_* (m/s)	*Humidity* (%RH)	*Icl_up_esti* (clo)	*Icl_lo_esti* (clo)	*Icl_esti* (clo)
winter	22.12	21.76	25.13	26.89	33.01	0.06	20.04	1.34	0.77	1.92
spring/fall	22.85	22.41	26.93	27.23	33.69	0.06	19.74	0.93	0.85	1.65
summer	23.40	22.79	29.45	27.28	33.85	0.07	18.94	0.46	0.93	1.32

**Table 8:** Layer properties table of upper body in simulation.

Layer No. (Chest)	Layer No. (Arm)	Material	Thickness (mm)	Thermal Resistance (m^2^·K/W)
19	18	Winter working jacket (company’s uniform)	10	0.085 *
18	17	shirts long sleeves shirt collar	1	0.048
17		Underwear shirts sleeveless	1	0.009
16~1	16~1	Body segments (skin, muscle, fat, bone *etc.*)	-	-

**Table 9:** Layer properties table of lower body in simulation.

Layer No. (Hip)	Layer No. (Leg)	Layer No. (Foot)	Material	Thickness (mm)	Thermal Resistance (m^2^·K/W)
		18	Shoes	1.5	0.003
		17	Socks	1	0.003
18	17		Trousers straight fitted	1	0.034
17			Underwear pants briefs	1	0.006
16~1	16~1	16~1	Body segments (skin, muscle, fat, bone *etc.*)	-	-

**Table 10:** Comparison of experiment and simulation according to clothing insulation values inputs concerning winter clothing ensembles, (Δ%error) = %error of Case 1 − %error of Case 2.

Simulation Model		Skin Temp (Face)				Clothing Temp			
						Chest		Legs	
		(°C)		%error (Δ%error)		(°C)	%error (Δ%error)	(°C)	%error (Δ%error)
IR camera measurement (Actual)		33.01		-		25.13		26.89	-
Case 1: Individual clothing insulation values without air layers between clothing		34.16		3.48		27.53	9.55	28.63	6.47
Case 2: Ensemble thermal insulation values of the estimated clothing insulation (*Icl_up_esti* = 1.34, *Icl_lo_esti* = 0.77)		34.37		4.12 (−0.64)		26.78	6.57 (2.98)	27.63	2.75 (3.72)
Case1:	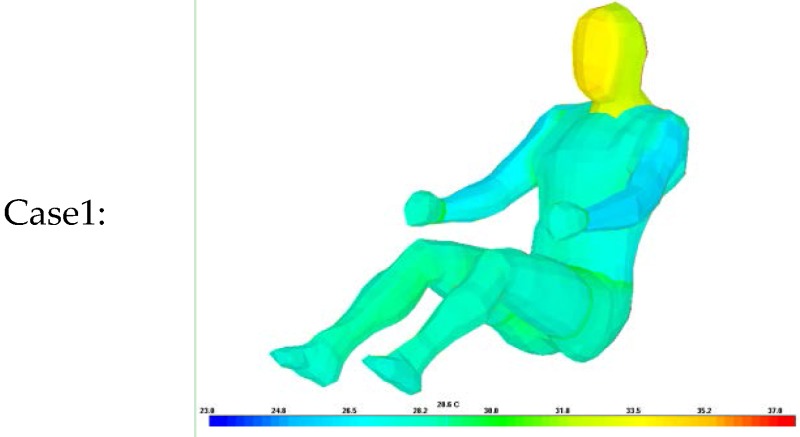		Case2:		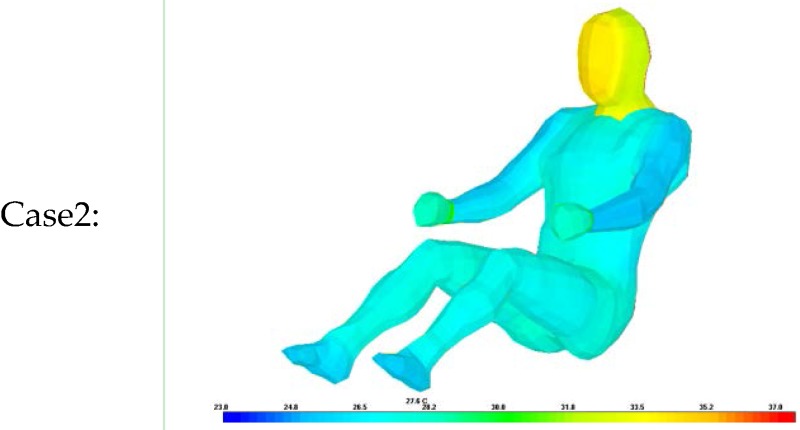				

**Table 11:** Comparison of experiment and simulation according to clothing insulation values inputs concerning spring/fall clothing ensembles, (Δ%error) = %error of Case 1 − %error of Case 2.

Simulation Model		Skin Temp (Face)				Clothing Temp.				
						Chest		Legs		
		(°C)		%error (Δ%error)		(°C)	%error (Δ%error)	(°C)	%error (Δ%error)	
IR camera measurement (Actual)		33.69		-		26.93		27.23	-	
Case 1: Individual clothing insulation values without air layers between clothing		34.43		2.20		28.42	5.53	29.04	6.65	
Case 2: Ensemble thermal insulation values of the estimated clothing insulation (*Icl_up_esti* = 0.93, *Icl_lo_esti* = 0.85)		34.60		2.70 (−0.5)		28.10	4.34 (1.19)	27.80	2.09 (4.56)	
Case1:	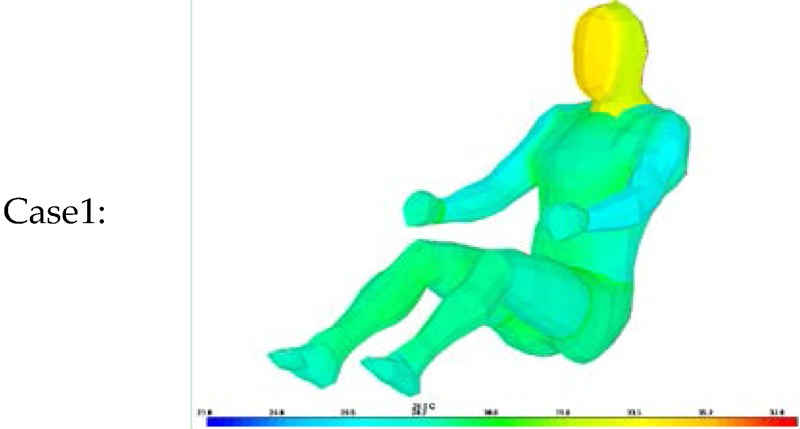		Case2:		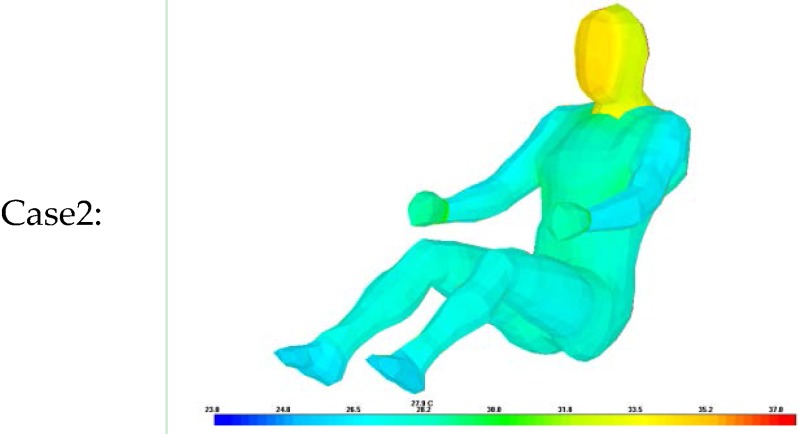				

**Table 12:** Comparison of experiment and simulation according to clothing insulation values inputs concerning summer clothing ensembles, (Δ%error) = %error of Case 1 − %error of Case 2.

Simulation Model	Skin Temp (Face)		Clothing Temp.			
			Chest		Legs	
	(°C)	%error (Δ%error)	(°C)	%error (Δ%error)	(°C)	%error (Δ%error)
IR camera measurement (Actual)	33.85	-	29.45		27.28	-
Case 1: Individual clothing insulation values without air layers between clothing	33.96	0.32	29.73	0.95	29.24	7.18
Case 2: Ensemble thermal insulation values of the estimated clothing insulation (*Icl_up_esti* = 0.46, *Icl_lo_esti* = 0.93)	34.09	0.71 (−0.39)	29.75	1.02 (−0.07)	27.55	0.99 (6.19)
Case1: 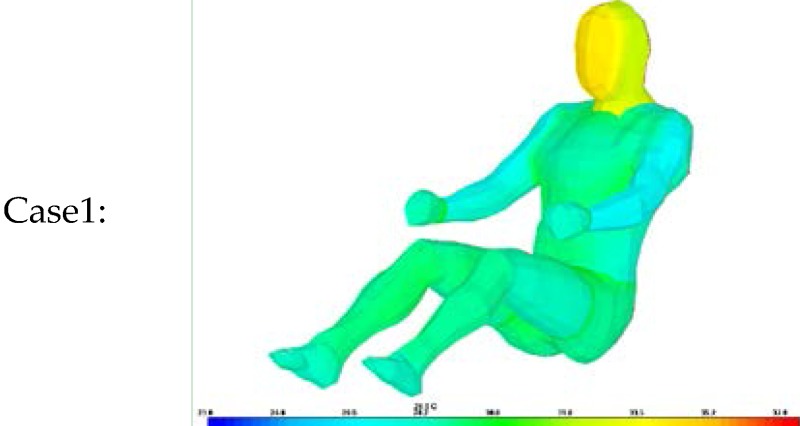	Case2:		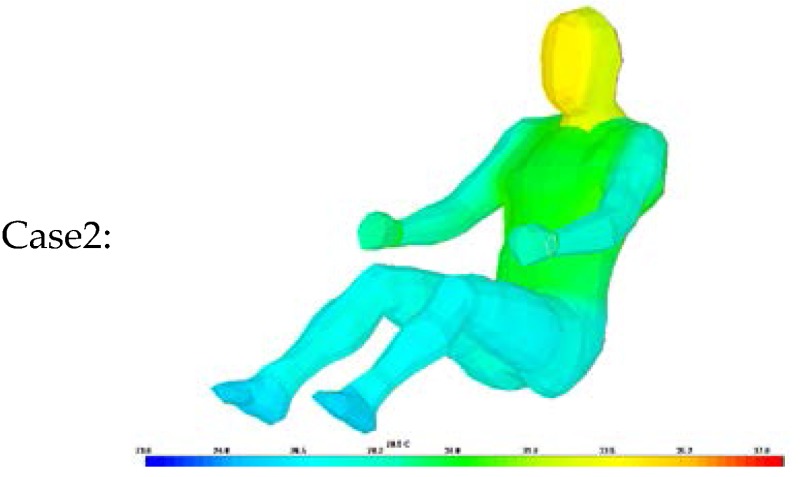			

**Table 13:** Clothing insulation effect testing with different values of air layer thickness (air velocity 0.02 m/s, humidity 24.7%RH, SD means standard deviation).

Air Layer (Thickness (mm))	Conditions		Visible Image	Thermal Image	Skin Temp, Face (*t_sk_*) (°C)	Cloth Temp, Abdomen (*t_cl_*) (°C)	*t_sk_ − t_cl_* (°C)	Estimated Upper *Icl* (clo)
	*t_r_* (°C)	*t_a_* (°C)						
Test 1: 0 layer (0 mm)	26.6 ± 0.1	26.7 ± 0.1	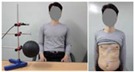	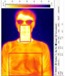	34.4 ± 0.1	30.6 ± 0.1	3.8	0.60
Test 2: 1 layer (3 mm)	26.3 ± 0.1	26.5 ± 0.1	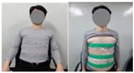	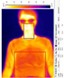	35.0± 0.1	30.2 ± 0.1	4.8	0.75
Test 3: 2 layer (6 mm)	26.2 ± 0.1	26.3± 0.1	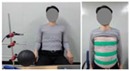	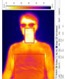	34.6 ± 0.1	29.5 ± 0.1	5.1	0.92

**Table 14:** Comparison of experiment and simulation for different air layer thicknesses.

Air Layer (Thickness (mm))	Skin (Face) Temp			Clothing (Chest) Temp		
	Actual (°C)	Simulation (°C)	Error (%error)	Actual (°C)	Simulation (°C)	Error (%error)
Test 1: Shirts and 0 air layer with estimated *I_cl_* = 0.6	33.1	33.3	0.4	29.8	29.9	0.2
Test 2: Shirts and 1 air layer with estimated *I_cl_* = 0.75	33.6	33.3	-0.9	29.3	29.5	0.7
Test 3: Shirts and 2 air layers with estimated *I_cl_* = 0.92	33.5	33.2	-0.8	28.4	28.9	1.9
